# Antifibrinolytic Agents: Aprotinin, and Desmopressin

**Published:** 2009-06

**Authors:** Pramila Bajaj

**Affiliations:** Editor, IJA

Antifibrinolytic agents are synthetic lysine analogs that include ε-aminocaproic acid (EACA, Amicar) and tranexanlic acid. These molecules inhibit fibrinolysis by attaching to the lysine binding site of the plasminogen molecule, displacing plasminogen from fibrin. Because fibrinolysis exhibits a major cause of bleeding, these agents have been reported to be effective in multiple surgical procedures^1^.

Desmopressin acetate (DDAVP) is a synthetic analog of vasopressin that increases plasma levels of factor VIII and stimulates vascular endothelium to release the larger multimers of von Willebrand factor(vWF)^2^. vWF mediates platelet adherence to vascular subendothelium by functioning as a protein bridge between glycoprotein Ib receptors on platelets and subendothelial vascular basement membrane proteins. DDAVP shortens the bleeding time of patients with mild forms of hemophilia A or von Willebrand disease (vWD)^2^,^3^. Which patients might benefit from use of DDAVP? Patients with mild to moderate forms of hemophilia or vWD undergoing surgery are likely to benefit from its use. In addition, patients with uremic platelet dysfunction and patients with chronic liver disease undergoing major surgery may benefit. Mongan and Hosking reported that patients with a thromboelastogram (TEG) taken after protamine administration and with maximal amplitude <50mm benefit from the effects of DDAVP^4^. DDAVP administered IV at a dose of 0.3 mg/kg achieves maximal increases in levels of factor VIII and vWF in 30-60 min with no further increases achieved by higher doses. It should be diluted and given over 15-30 min to avoid hypotension. Side effects include hyponatremia with repeated dosing. Unfortunately, most studies do not demonstrate consistent efficacy of DDAVP^5^.

Aprotinin is a broad-spectrum serine protease inhibitor that inhibits factor XII, kallikrein, plasmin, and PAR1 receptors^6^. In cardiac surgery when used prophylactically, multiple randomized placebo-controlled trials on aprotinin safety and efficacy have demonstrated that aprotinin therapy reduces bleeding (i.e., mediastinal and chest tube drainage) and decrease^3^ the need for allogeneic transfusion, and the proportion of patients needing transfusion of allogeneic blood^7^,^8^.

Sedrakyan reported data from 35 CABG trials (n=3879) confirming that aprotinin reduces transfusion requirements (relative risk 0.61) relative to placebo, with a 39% risk reduction, and was not associated with increased or decreased mortality (relative risk 0.96), myocardial infarction (relative risk 0.85), or renal failure (relative risk 1.01) risk, but it was associated with a reduced risk of stroke (relative risk 0.53). Aprotinin's mechanism of action is complex and may also involve reduction of the inflammatory response^9^. Multiple mechanisms are responsible for aprotinin's ability to reduce bleeding after CPB and in other surgical procedures.

Aprotinin has also been studied in clinical trials in vascular, liver transplantation^10^, and orthopedic surgery^1^. Aprotinin decreased intra- and postoperative bleeding and blood transfusion in these settings. In orthopedic surgery, aprotinin moderately decreases blood loss and transfusion requirements during total hip replacement. One or two packed red cell units per patient may be saved when aprotinin is used. In a double-blind study in high-risk septic and cancer patients undergoing pelvic and hip surgery, aprotinin proved to be effective in significantly reducing the need for blood transfusion as compared with a placebo group. Samama et al evaluated two doses of aprotinin with placebo after major orthopedic surgery and reported blood loss decreased in the Large-Dose Aprotinin group (calculated bleeding, whole blood, hematocrit 30%, median [range], 2023 ml, [633-4113] as compared with placebo, 3577 mL [1670-21, 758 mL]. The total number of homologous and autologous units was also significantly decreased in the Large-Dose Aprotinin group (2 U [0-5 U] as compared with placebo, 4 U [0-42U])^11^

## References

[1] Zuferey P, Merquiol F, Laporte S (2006). Do antifibrinolytics reduce alloeeneic blood transfusion in orthopedic surgery. Anesthesiology.

[2] Mannucci PM (2004). Treatment of von Willebrand's Disease. N Engl J Med.

[3] Mannucci PM (1998). Hemostatic drugs. N Engl J Med.

[4] Mongan PD, Hosking MP (1992). The role of desmopressin acetate in patients undergoing coronary artery bypass surgery. A controlled clinical trial with thromboelastographic risk stratification. Anesthesiology.

[5] de Prost D, Barbier-Boehm G, Hazebroucq J (1992). Desmopressin has no beneficial effect on excessive postoperative bleeding or blood product requirements associated with cardiopulmonary bypass. Thromb Haemost.

[6] Landis RC, Asimakopoulos G, Poullis M (2001). The antithrombotic and antiinflammatory mechanisms of action of aprotinin. Ann Thorac Surg.

[7] Royston D, Levy JH, Fitch J (2006). Full-dose aprotinin use in coronary artery bypass graft surgery: an analysis of perioperative pharmacotherapy and patient outcomes. Anesth Analg.

[8] Sedrakyan A, Wu A, Sedrakyan G (2006). Aprotinin use in thoracic aortic surgery: safety and outcomes. J Thorac Cardiovasc Surg.

[9] Mojcik CF, Levy JH (2001). Aprotinin and the systemic inflammatory response after cardiopulmonary bypass. Ann Thorac Surg.

[10] Porte RJ, Molenaar IQ, Begliomini B (2000). Aprotinin and transfusion requirements in orthotopic liver transplantation: a multicentre randomised double-blind study. EMSALT Study Group. Lancet.

[11] Samama CM, Langeron 0, Rosencher N (2002). Aprotinin versus placebo in major orthopedic surgery: a randomized, double-blinded, dose-ranging study. Anesth Analg.

